# Robotic-Assisted Retroperitoneal Lymph Node Dissection in a Challenging Post-Chemotherapy Case

**DOI:** 10.1590/S1677-5538.IBJU.2025.0466

**Published:** 2025-09-10

**Authors:** Willy Baccaglini, Gabriel Chahade Sibanto Simões, Julio Calderón, Nicolle Christofe, Luis G. Medina, Paulo Priante Kayano, Gustavo Lemos, Arie Carneiro

**Affiliations:** 1 Faculdade de Medicina do ABC Departamento de Urologia Santo André SP Brasil Departamento de Urologia, Faculdade de Medicina do ABC, Santo André, SP, Brasil; 2 Hospital Israelita Albert Einstein Serviço de Urologia São Paulo SP Brasil Serviço de Urologia, Hospital Israelita Albert Einstein, São Paulo, SP, Brasil; 3 Departamento de Oncologia do Hospital Beneficência Portuguesa de São Paulo São Paulo SP Brasil Departamento de Oncologia do Hospital Beneficência Portuguesa de São Paulo, São Paulo, SP, Brasil; 4 Department of Urology Medical University of South Carolina Charleston South Carolina United States Department of Urology Medical University of South Carolina, Charleston, South Carolina, United States

## Abstract

**Introduction:**

Robotic retroperitoneal lymph node dissection (RPLND) has emerged as a minimally invasive alternative for the management of testicular germ cell tumors, offering reduced morbidity and faster recovery when performed in experienced centers (1–3). However, post-chemotherapy cases remain technically demanding. We present a case of robotic RPLND performed for a bulky residual mass following systemic treatment.

**Methods:**

A 23-year-old male with no comorbidities underwent right orchiectomy for clinical stage IIC non-seminomatous germ cell tumor (60% yolk sac, 20% embryonal carcinoma, 20% post-pubertal teratoma), followed by three cycles of BEP chemotherapy. Tumor markers normalized, but imaging revealed a persistent 5.4-cm interaortocaval mass. Robotic RPLND was carried out using four robotic ports and one 12-mm assistant port. The procedure included a complete bilateral template dissection (paraaortic, interaortocaval, and paracaval), en bloc tumor removal, and meticulous sharp and blunt dissection using advanced bipolar energy.

**Results:**

Operative time was 300 minutes, with minimal blood loss (50 mL) and no intraoperative complications. The bulky lesion was successfully resected with excellent anatomical exposure, despite significant tumor adherence to the aorta. The patient was discharged on postoperative day one and resumed normal activities within two weeks. Pathology revealed teratoma in 1 of 34 resected lymph nodes. At 6-month follow-up, he remained disease-free, with normal tumor markers, preserved renal function, and no complications.

**Conclusion:**

This case demonstrates the feasibility of robotic RPLND for large post-chemotherapy residual masses. The robotic platform enables precise dissection even in challenging settings, with favorable perioperative and oncologic outcomes. Centralized expertise and standardized technique are essential to achieve optimal results (1, 4–6).

**Available at:**VIDEO

## Data Availability

Uninformed
